# Protective Role of Vitamins C, D, and K in the Management of Dengue: A Narrative Review to Combat the Dengue Outbreak

**DOI:** 10.1002/hsr2.71823

**Published:** 2026-02-17

**Authors:** Shah Md Muztahid Hasan Chowdhury, Md. Shafiul Hossen, Mohammad A. Rashid, Mahammed Shafin Ul Islam, Arafat Miah, Mohammed Safiqul Islam, Md. Abdul Barek

**Affiliations:** ^1^ Department of Pharmacy Noakhali Science and Technology University Noakhali Bangladesh; ^2^ Department of Pharmacy State University of Bangladesh Dhaka Bangladesh; ^3^ Department of Pharmaceutical Chemistry Faculty of Pharmacy University of Dhaka Dhaka Bangladesh; ^4^ Department of Pharmacy Southern University Bangladesh Chattogram Bangladesh

**Keywords:** dengue, management, outbreak, protective role, vitamin C, D, and K

## Abstract

**Background and Aims:**

Dengue fever is mostly found in tropical and subtropical regions and is spread by mosquitoes. Still now, no specific antiviral medication is available against the disease. However, several vitamins have been identified as potential dengue therapy supplements. Therefore, this study aimed to comprehensively gather data on the protective role of vitamins C, D, and K in the treatment of dengue fever.

**Methods:**

To conduct the present narrative review, we retrieved data from Google Scholar, Web of Science, PubMed, Cochrane Library, BanglaJOL, Embase, and Scopus regarding the influence of vitamin supplements in dengue treatment. Articles published in English before March 2025 were included in the study.

**Results and Discussion:**

This review explored how vitamin C neutralizes reactive oxygen species and reduces inflammation and oxidative damage in dengue infection. It stimulates chemotaxis and NK cell activity and controls the activities of monocytes, macrophages, phagocytosis, and the release of cytokines that promote inflammation. While shielding neutrophils from oxidative damage, it also promotes ROS production, which is essential for pathogen removal. Additionally, vitamin D possesses antiviral and immunomodulatory properties that help to fight dengue virus (DENV). It regulates the course of the disease in DENV‐infected individuals by modifying immunological responses. It can limit viral replication, decrease the expression of DENV entry receptors, and alter the expression of inflammatory cytokines in cells infected with the virus. Moreover, vitamin K is recommended in cases of dengue‐related hepatic failure and encephalopathy. The administration of vitamin K in early dengue hemorrhagic fever (DHF) may help to avoid anemia and lessen the need for transfusions.

**Conclusion:**

By targeting important pathogenic processes, the adjunctive use of vitamins C, D, and K together provides an effective supplemental strategy in the dengue management and provides a good patient outcome in future dengue outbreak.

## 1 Introduction

Dengue is a viral disease that is expanding globally and is especially common in tropical countries, which is spread by female *Aedes* mosquitoes and carried by any of the four serotypes [1]. Dengue poses a threat to about one‐third of the world's population [1]. Currently, dengue is prevalent in 128 countries, mostly in developing countries, and poses a yearly threat to over 3.97 billion people [2]. According to a new dengue distribution model, there are 390 million dengue infections each year, out of which 96 million cases appear to be clinically significant [3].

From moderate asymptomatic dengue fever (DF) to severe dengue hemorrhagic fever (DHF) and possibly lethal dengue shock syndrome (DSS), dengue virus (DENV) infection results in a variety of clinical manifestations, including a sudden high temperature, headache, muscle and joint discomfort, rash, and minor bleeding, which in severe cases can proceed to plasma leakage, severe hemorrhage, thrombocytopenia, and periodic shock [4]. Since there is not a particular antiviral drug available yet, this disease is still challenging to treat. That's why supportive care is the basis of dengue management to reduce symptoms and avoid complications. Although Dengvaxia, a commercial vaccine, is available in certain dengue‐endemic nations, its effectiveness varies depending on the DENV serotype [5]. Concerns have also been raised over the possibility of more severe illness in those who have never been exposed to flaviviruses before after getting the vaccination [6]. While several medications have started small‐scale trials as prospective therapies, the majority of candidate medications have not demonstrated a significant reduction in participant viremia [7, 8].

The possible antiviral activity of micronutrient status and supplementation in the host has been extensively researched over the past decade, including vitamins C, K, and D and they have been studied for their potential to enhance immune function and provide antiviral benefits [5]. The complex interactions between the virus, host genes, and the host's immune response are believed to cause the pathophysiology of DENV infection, with the host's immune response playing a major role. When the infection is being eradicated by the host immune response, DENV infection really shows up as the severe form of the illness, which is not correlated with the peak viral load [9]. After invading immune cells, it causes endothelial dysfunction, increased vascular permeability, and immunological dysregulation [10]. Additionally, oxidative stress is also linked to the progression of the disease, it exacerbates vascular damage and immunological activation [11].

Nutrition is one of several elements that affect the immune system and its competence. There is an inverse relationship between immunity, infection, and nutrition; modifications to one factor have an impact on the others [12]. By compromising immunological responses and intensifying oxidative stress, deficiencies in vital vitamins and minerals may increase the severity of disease [12]. However, numerous vitamins have been recognized as possible supplements for the treatment of dengue. Therefore, we aimed to explore the role of vitamins C, K, and D in treating dengue and preventing serious complications related to this disease. This review focused on the protective role of vitamins C, D, and K in managing dengue outbreak. The purpose of this study was to highlight their therapeutic value in dengue treatment by examining their possible effects on oxidative stress, vascular integrity, coagulation, and immunological modulation. Furthermore, the results might provide information on novel dietary approaches that enhance existing clinical treatments and enhance more study in this area.

## 2 Methods

### 2.1 Data Searching

Information about the contributing factor on dengue susceptibility and function of vitamins in treating dengue was obtained from several web databases such as PubMed, Google Scholar, Embase, Web Sciences, and the Cochrane, and so forth. We retrieved articles in English that were published before March 2025. To gather information, we employed a number of terms, such as “Dengue,” “Infection/Contagious diseases/Outbreak,” “Epidemiology/Prevalence/Statistics of Dengue,” “Vitamin/Vitamin K/Vitamin C/Vitamin D,” and “Role/Impact/Management/Treatment.” In addition, to identify any pertinent missing articles, we looked through the references of the chosen articles and reviews that were written about this topic.

### 2.2 Eligibility Criteria

When extracting data from the chosen articles, a number of characteristics were considered. The following eligibility criteria were met by the studies that were included in this study: (a) Articles about dengue infection, (b) Studies on the role of vitamins in managing dengue, (c) Articles on dengue treatment, and (d) Articles that were published in English. However, the present study excluded some studies based on the predetermined exclusion criteria such as: (a) Studies published in local language, (b) Editorials and comments, (c) Papers with inadequate information about the effect of vitamins on dengue management, and (d) Articles that were restricted to access.

### 2.3 Data Extraction

Two separate researchers (S. M. M. H. C. and M. S. H.) independently screened and extracted data, employing the inclusion and exclusion requirements, and any disagreements were resolved through detailed discussion. Basic details of the included papers were noted, including the name of the author, study title, abstract, and findings. During the data extraction procedure (Figure S1), the results of the chosen publications, the study's eligibility requirements were all considered. We prepared a checklist containing the fundamental characteristics of the included studies to ensure the reliability of the data. The checklist included a clear statement of the aims of the research, inclusion criteria, methodology, research design, sources of data, ethical issues, clear statement of findings, and so forth (Table S1). Studies that did not meet the expected data collection requirements, they were excluded from the current study. Information from the included studies was presented regarding the pathophysiology of dengue and oxidative stress to dengue complications. This study also demonstrated the protective role of specific vitamins (C, D, and K), highlighting their respective biological mechanisms and clinical relevance in dengue management. Finally, we retrieved data from studies comprising in vitro (*n* = 8), observational (*n* = 17), animal (*n* = 3), clinical studies (*n* = 2), and other literature to explore the role of vitamins C, D, and K in the dengue management.

## 3 Results and Discussion

### 3.1 Pathophysiology of Dengue

Although the initial targets are believed to be skin macrophages and dendritic cells, it is still unclear what happens if a mosquito bite spreads the DENV to the skin. These affected immune cells are believed to reach the lymph nodes and then spread to other organs via the lymphatic system [13]. Immune‐mediated consequences after DENV infection and the pathophysiology of the infection are continuously evolving. The primary viral protein (E) glycoprotein, which binds with host cells, mediates the critical stage of virus infection [14]. An initial infection with the same serotype frequently results in lifetime immunity to infection with that serotype. Afterwards, cross‐reactivity between virus proteins and serotypes may trigger a secondary immune response, which can lead to consequences during a secondary infection, such as organ failure [13, 15].

**Figure 1:**
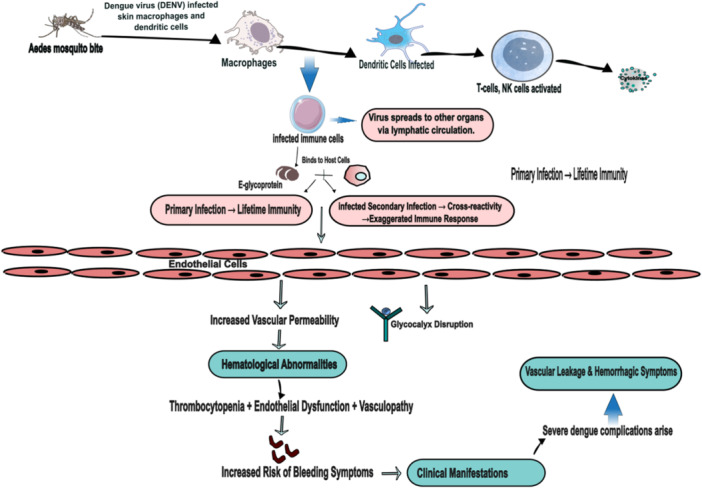
Pathophysiology of dengue infection.

Endothelial/vascular permeability and glycocalyx alteration brought on by toxic mediators are the causes of this pathophysiologic dysfunction [13, 15]. The most important ones are free vascular endothelial growth factor, IL‐6, IFN‐gamma, and nitric oxide. Clinical symptoms and problems in dengue are caused by a complicated interaction between several biochemical mediators and the endothelium [15]. However, increased levels of Von Willebrand factor, tissue factor, tissue plasminogen activator, and platelet factor inhibitor are among the most common hematological abnormalities. Thrombocytopenia, endothelial dysfunction, and vasculopathy all work together to increase the risk of bleeding symptoms [15, 16]. Figure 1 shows the pathophysiology of dengue infection [17].

### 3.2 Oxidative Stress to Dengue Complications

Reactive oxygen species (ROS) are produced in excess when infected by the DENV, mostly via mitochondrial pathways through stimulating NADPH oxidase. Consequently, endothelial cells get damaged by this ROS, which increases vascular permeability and disrupts the barrier [18, 19]. The immunological response to DENV is characterized by a “cytokine storm,” which is a rise in pro‐inflammatory cytokines [20]. Furthermore, vascular permeability is increased in severe dengue. It is caused by immune‐mediated elements like histamine and inflammatory cytokines as well as direct viral effects on endothelial cells. Subsequently, these elements interfere with endothelial junctions, which allows plasma to leak, which is a major contributing factor to shock and hemorrhagic complications [18, 19].

### 3.3 Roles of Vitamins C, D, and K in Dengue Management

#### 3.3.1 Vitamin C

**Figure 2:**
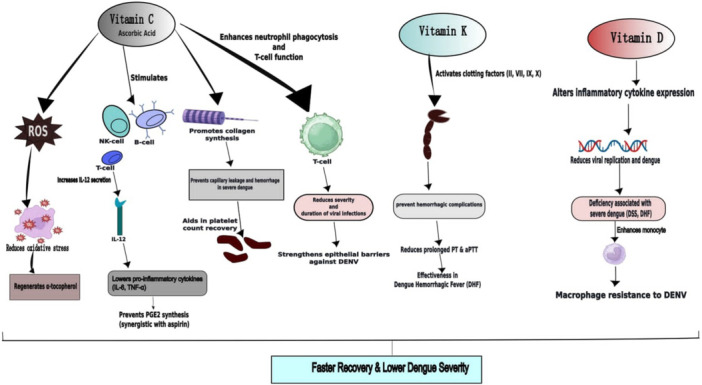
Protective role of vitamins C, D, and K in dengue infection.

Vitamin C is a vital water‐soluble micronutrient required for many biological processes. It promotes healthy growth and development and serves as an essential enzyme cofactor in a number of events, such as the synthesis of collagen, the manufacture of carnitine, the conversion of dopamine to norepinephrine, the metabolism of tyrosine, and so forth. It also promotes iron absorption and various infection prevention [21]. The effect of vitamin C as a supportive treatment for dengue is shown in Tables 1, 2, and Figure 2.

**Table 1:** Effect of vitamins C, D, and K in the dengue management.

Vitamin	Study type	Study design	Findings	Limitations	Strength of evidences	Ref.
Vitamin C	Retrospective study (India)	Double‐blind, placebo‐controlled	Higher platelet count and shorter hospital stays in supplemented groups.	No inclusion of oxidative stress biomarkers. Follow‐up period was limited to 5 days.	Randomized controlled trial design. Implementation of standardized treatment protocols.	Karthik et al. [22]
	Randomized Controlled Trial (India)	NA	Platelet counts increased in the vitamin C group. Faster platelet recovery and fewer bleeding complications.	NA	NA	Langerman and Ververs [23]
Vitamin K	Retrospective study (Niloufer Hospital, Hyderabad)	Cases study	Positive responses were observed. Within 24 h of medication, the bleeding ceased.	No serial monitoring of clinical and laboratory data.	WHO guidelines were used to define clinical and confirmed DHF cases.	Srigade [24]
Vitamin D	Observational study	Case control study	Lower serum vitamin D levels linked to severe dengue.	NA	NA	Giraldo et al. [25]
	Exploratory study	Prospective, single‐center, single‐group, cross‐sectional study	Lower pro‐inflammatory cytokine levels were observed. Patients with higher vitamin D levels were less prone to DENV infection. Higher doses (4000 IU/day) led to greater resistance.	Lack of control group or healthy volunteers. Small sample size.	Assay by ELISA technique.	Iqtadar et al. [26]
	Observational study	Cases study	Positive responses at higher vitamin D levels in dengue patients, particularly in severe cases.	No serotypes in the study population. No secondary infection patient.	WHO guidelines were used to define clinical and confirmed DHF cases. Laboratory investigations.	Bharara et al. [27]

**Table 2:** A summary table comparing the effects and limitations of vitamins C, D, and K across study types.

Vitamin	Effects	Limitations
Vitamin C	Neutralizes reactive oxygen species (ROS) and lipid radicals; regenerates α‐tocopherol. Enhances T and B cell activity, NK cell function, chemotaxis, and IL‐12 secretion; reduces TNF‐α, IL‐6, and CRP levels. Lowers NF‐κB activation and PGE_2_ synthesis. Promotes collagen synthesis, improving vascular integrity and reducing plasma leakage.	Dose–response relationships unclear. Lack of long‐term safety/efficacy data.
Vitamin D	Regulates cytokine production (↓ TNF‐α, IL‐6; ↑ IL‐10), enhances SOCS protein synthesis, and modulates TLR3/TLR9 pathways. Reduces dengue virus replication and downregulates viral entry receptors.	Small sample sizes and heterogeneous populations. Lack of randomized controlled trials. Findings not generalizable to all populations.
Vitamin K	Serves as a cofactor for hepatic carboxylation of clotting factors II, VII, IX, and X. Shown to reduce bleeding duration and transfusion requirement in DHF. Recommended in national guidelines (e.g., Sri Lanka) for dengue‐related hepatic failure with prolonged PT.	Lack of large‐scale or controlled trials. Effect limited to cases with hepatic dysfunction or coagulopathy. Routine use not recommended by WHO.

#### 3.3.2 Antioxidant Role

By neutralizing lipid hydroperoxyl radicals, scavenging ROS, and protecting proteins from alkylation by electrophilic lipid peroxidation products, vitamin C contributes to the prevention of oxidative stress‐induced cellular damage [28]. Its antioxidant mechanism of action is centered on quenching singlet oxygen, removing molecular oxygen, and providing lipid radicals with a hydrogen atom. It also includes aqueous radical scavenging and α‐tocopherol regeneration from tocopherol radical species [29]. These mechanisms provide biological plausibility for a potential role of vitamin C in modulating oxidative stress and inflammatory pathways relevant to dengue; however, direct clinical evidence demonstrating reduced inflammation or oxidative damage in dengue patients remains limited and primarily observational [28].

#### 3.3.3 Immunomodulatory and Anti‐Inflammatory Effects

Normal immune defense requires an adequate basal concentration of vitamin C. Experimental and mechanistic studies indicate that Vitamin C stimulates several components of innate and adaptive immunity, including stimulation of chemotaxis, lymphocyte proliferation, and NK cell activity. High dosages of it have been shown to activate T and B cell functions and promote IL‐12 secretion, largely based on non‐dengue models [30]. Vitamin C also controls the activities of monocytes and macrophages, improves phagocytosis, and lowers the release of cytokines that promote inflammation, such as TNF‐α and IL‐6. In neutrophils, it supports mobility and chemotaxis, protects against oxidative damage, and facilitates oxygen species generation required for pathogen clearance [31].

Through the modulation of multiple inflammatory pathways, vitamin C exhibits notable anti‐inflammatory effects. A review suggests that vitamin C supplementation may be associated with lower circulating levels of inflammatory markers, including C‐reactive protein (CRP), interleukin‐6 (IL‐6), and NF‐κB/TNF‐α signaling [32]. In a study of obese adults with hypertension and/or diabetes, it was shown that taking 500 mg of vitamin C twice a day for 8 weeks significantly decreased these inflammatory markers [33]. However, such findings derive from noninfectious clinical contexts and cannot directly extrapolated to dengue infection.

In addition, vitamin C functions as an antioxidant by scavenging ROS, which may limit tissue injury associated with inflammatory responses [32]. Experimental studies also suggest that vitamin C can inhibit prostaglandin E_2_ (PGE_2_) synthesis and may enhance the inhibitory effects of aspirin on PGE_2_ production in neuronal cell models, indicating a potential synergistic mechanism in inflammation regulation [34].

#### 3.3.4 Vascular Integrity and Collagen Synthesis Role

Vitamin C is crucial for collagen synthesis, which is vital for maintaining vascular integrity. It serves as a cofactor for the hydroxylation enzyme, which is necessary for the stability and maturation of collagen fiber [35]. Moreover, a review indicates that vitamin C promotes type IV collagen synthesis and deposition in the endothelium, leading to vascular stability and function. Consequently, it may aid in preventing capillary leakage and lower the chance of hemorrhage and other associated problems by strengthening the collagen structures of the vascular system [36]. As plasma leakage is a complication of dengue [16], by promoting collagen synthesis, vitamin C can reduce this complication in DHF. A randomized controlled trial revealed that vitamin C can be used as adjuvant therapy alongside standard dengue management (i.e., rehydration and antipyretics) to improve not only the platelet counts but also expedite recovery without a notable increase in adverse events (renal oxalate, G6PD deficiency) [22].

#### 3.3.5 Antiviral Activities

Vitamin C has been investigated for potential antiviral and immunomodulatory effects across a range of viral infections, with most supporting evidence derived from mechanistic studies, animal models, and non‐dengue clinical contexts. Experimental data suggest that vitamin C contributes to immune regulation by facilitating the clearance of accumulated neutrophils through macrophage‐mediated apoptosis and phagocytosis, processes that are important for T‐cell maturation during viral infections [37]. In murine models, vitamin C supplementation has been associated with improved survival in several viral infections [38]. For example, Valero and colleagues reported increased survival in mice infected with Venezuelan equine encephalitis virus following vitamin C administration [39]. Similarly, influenza‐infected mice unable to synthesize vitamin C exhibited greater lung pathology in the absence of supplementation, indicating a protective role in this experimental setting [40]. Thus, high doses of vitamin C in animal and experimental settings have demonstrated the ability to reduce both the duration and severity of viral infections like influenza and herpes simplex virus [37]. It can also reduce the mortality rate in H1N1 virus‐induced pneumonia [41]. However, these findings are largely limited to animal models or non‐dengue viral infections and cannot be directly extrapolated to clinical efficacy in humans. Emerging experimental and observational evidence also suggests that vitamin C may enhance epithelial barrier function and support innate immune responses, including neutrophil activity, which may be relevant to host defense against DENV and other pathogens [23, 42, 43, 44]. Overall, these data support biological plausibility for an immunomodulatory role of vitamin C in viral infections; however, clinical evidence demonstrating antiviral efficacy or reduced inflammation in dengue remains limited and low certainty.

#### 3.3.6 Vitamin K

In dengue, spontaneous bleeding is unlikely due to thrombocytopenia. A coagulopathy‐related thrombocytopenia makes the bleeding more severe. Common hematological abnormalities include an abnormal coagulation profile, thrombocytopenia, platelet dysfunction, liver‐related prothrombin complex deficiency, endothelial injury, prolonged activated partial thromboplastin time, and disseminated intravascular coagulation (characterized by decreased fibrinogen levels, increased fibrinogen degradation products, elevated D‐dimer levels, consumptive coagulopathy, and activation of mononuclear phagocytes) [45]. Bleeding can manifest as hematuria, hematemesis, gingival bleeding, melena, epistaxis, and spontaneous skin bleeding. Depending on the patient's state and the availability of supportive care like fresh frozen plasma, platelets, or packed cell transfusions, they may be administered in cases of severe bleeding with coagulopathy [46]. In conjunction with these interventions, intravenous vitamin K should be considered in cases of bleeding. Prothrombin time (PT) and aspartate aminotransferase/alanine aminotransferase levels may be elevated in some individuals with hepatic injury. In such circumstances, intravenous vitamin K may be initiated if PT is extended [45, 46].

A microsomal enzyme that catalyzes the posttranslational carboxylation of several distinct, peptide‐bound glutamic acid residues in inactive hepatic precursors of factors II, VII, IX, and X requires vitamin K as a necessary cofactor [47]. Although the WHO added vitamin K to the necessary drugs list for dengue management in its guidelines, but it should not be routinely administered [48]. The guidelines mentioned the use of intravenous vitamin K1 in the management of dengue patients with hepatic dysfunction, especially if the PT is prolonged [48]. In dengue, an increased PT denotes bleeding brought on by liver failure. An epidemiological study showed that the national guidelines of Sri Lanka recommended to use of vitamin K in cases of dengue‐related hepatic failure and encephalopathy [49]. A retrospective study conducted in Hyderabad also assessed the effectiveness of vitamin K in patients who were admitted with confirmed and clinical DHF. This study proved the clinical effectiveness of vitamin K as the fact that the bleeding stopped within 24 h of the supplement's administration and did not return. Additionally, vitamin K given early in DHF may help to avoid anemia and lessen the need for transfusions [24]. The impact of vitamin K as a supportive treatment for dengue is highlighted in Table 1 and Figure 2. Moreover, Table 2 presents a summary table comparing the effects and limitations of vitamins K across study types.

#### 3.3.7 Vitamin D

There are no registered antiviral medications for dengue infections now. Therefore, people who have previously been exposed to the virus utilize a vaccination called Dengvaxia [5]. Immunomodulators that reduce inflammation are being researched as potential treatments for severe dengue because they prevent vascular leakage [50]. Because of its dual function, vitamin D can influence the immune response and have antiviral properties; it has emerged as a promising option among these. Moreover, numerous positive benefits have been shown in in‐vitro research using the active form of vitamin D [50]. This substance has been demonstrated to inhibit the DENV's ability to replicate, alter the production of inflammatory cytokines in infected cells, and reduce the expression of receptors that the virus utilizes to enter cells. However, this in‐vitro study also emphasizes the significance of maintaining appropriate levels for a protective impact by warning that insufficient vitamin D intake may potentially promote virus replication [50]. Due to the strong immunomodulatory effects of some nutrients, the nutritional condition of the host has been suggested as a potentially significant predictor of the course of the disease in dengue patients before treating with vitamin D [44]. By modifying toll‐like receptors, specifically TLR3 and TLR9, vitamin D offers clinical advantages and lessens harmful inflammatory reactions. Furthermore, it promotes the development of suppressor of cytokine signaling (SOCS) proteins and raises the synthesis of the anti‐inflammatory cytokine like Interleukin‐10 (IL‐10), both of which help to reduce excessive immune activation [23]. Available evidence indicates that vitamin D‐based therapies may offer potential benefits in dengue management, providing a promising outlook for patient treatment; however, these findings need to be confirmed through positive outcomes in clinical trials.

There is growing evidence associating vitamin D deficiency (VDD) to an increased risk of developing severe dengue fever. However, limited studies examined the possible relationship between VDD and dengue illness severity and susceptibility to severe dengue [25, 51, 52, 53, 54]. Vitamin D has been investigated for its potential impact in regulating the intensity of dengue fever in critically ill patients. A prospective cross‐sectional study found that lower serum vitamin D levels are linked to severe dengue, including DSS and DHF. This study also discovered a possible connection between elevated vulnerability to severe DF disease and serum VDD. In addition, lower median vitamin D levels are linked to more severe dengue infections [26]. Monocyte‐derived macrophages (MDMs) that differentiated with vitamin D not only expressed lower levels of mannose receptor but also produced fewer pro‐inflammatory cytokines and were less prone to DENV infection than MDMs that differentiated without vitamin D [25].

Due to its involvement in multiple important molecular pathways, vitamin D supplementation is especially relevant in DHF patients. It exerts its role in cellular mechanisms and autophagy regulation [55]. The intracellular calcium–calmodulin complex is the first thing that vitamin D interacts with when it enters the cell. Mammalian target of rapamycin (mTOR), a crucial regulator that typically inhibits autophagy, is inhibited by Ca^2+^–calmodulin complex; as a result, autophagy is promoted. This vitamin D–VDR complex then pairs with the retinoid X receptor (RXR) and migrates into the nucleus, the complex attaches to the vitamin D receptor element (VDRE) on DNA inside the nucleus, initiating the transcription of genes that include the DNA‐damage‐inducible transcript, which further slows down mTOR activity. The synthesis of light chain‐3 (LC3), a protein that both signals and stimulates autophagy, is also encouraged by this complex [55]. Then, protein disulfide‐isomerase‐A3 (PDIA3) is simultaneously bound by vitamin D, which promotes its interaction with the signal transducer and activator of transcription‐3 (STAT3). Because the DENV usually cleaves STAT3 to disrupt cellular functions, vitamin D's interaction with PDIA3 helps to create the autophagy‐supporting protein mucolipin‐3 (MCOLN3) in addition to protecting STAT3 from cleavage [55].

According to a previous research, macrophages derived from monocytes from healthy donors that received higher doses of vitamin D (4000 IU/day) produced much more IL‐10 and fewer pro‐inflammatory cytokines, and they were also more resistant to DENV‐2 infection [25]. The pathophysiology of dengue infection may be influenced by 25‐hydroxy vitamin D3, according to another study; as a result, vitamin D levels may be a useful prognostic indicator for forecasting the course of the dengue illness. This study found that dengue cases had noticeably higher levels of 25‐hydroxy vitamin D3 compared to healthy controls. Specifically, the levels were 1.26 times higher in dengue cases and 1.6 times higher in severe dengue cases compared to healthy controls [27]. These observed associations may reflect residual confounding related to baseline nutritional status, socioeconomic conditions, or environmental exposures rather than a direct therapeutic effect. Therefore, therapeutic efficacy should not be inferred in the absence of well‐designed randomized controlled trials. The impact of vitamin D as a supportive treatment for dengue is highlighted in Table 1 and Figure 2. Furthermore, Table 2 demonstrates a summary table comparing effects and limitations of vitamins C, D, and K across study types.

A few limitations of this study should be taken into account when interpreting the findings. First, the findings may not be broadly applicable due to its limited clinical data. Second, the data might not accurately reflect the overall population because the studies included in the study had limited sample size. Third, potential confounding variables that might have affected serum vitamin D levels, such as dietary status, sun exposure, and previous vitamin D supplementation, were not considered. For instance, what if certain individuals have varying nutritional statuses, take vitamin D supplements or receive more sun exposure, which may raise vitamin D levels. These elements may have an impact on vitamin D levels. Finally, we included limited studies due to the lack of sufficient studies regarding the role of vitamins C, D, and K in dengue management. If we found more studies, it would provide the more accurate outcome.

## 4 Conclusions

This narrative review highlights the potential adjunctive roles of vitamins C, D, and K in dengue management through complementary mechanisms. Vitamin C may attenuate oxidative stress and endothelial injury, vitamin D exerts immunomodulatory and antiviral effects, and vitamin K supports coagulation and vascular stability. Collectively, these actions suggest a possible role in reducing the risk of progression to severe dengue. However, the overall certainty of the current evidence remains low. Most available data are derived from observational studies, in‐vitro experiments, and limited clinical investigations, with a clear lack of well‐powered randomized controlled trials. In addition, there is no standardized approach to vitamin supplementation in dengue, and key factors such as baseline nutritional status, disease phase, and population‐specific characteristics are often not considered. The underlying molecular mechanisms, particularly those related to viral entry inhibition, cytokine modulation, and vascular protection, also remain incompletely understood.

Future research should therefore prioritize on dose–response randomized controlled trials to establish the efficacy, safety, and optimal dosing regimens of vitamins C, D, and K; phase‐specific supplementation studies to determine whether benefits differ across febrile, critical, and recovery phases of dengue; and population‐focused trials, particularly in pediatric, pregnant, and malnourished populations in endemic settings. Additionally, dedicated controlled trials evaluating vitamin K supplementation in severe dengue are warranted to clarify its role in reducing bleeding risk, anemia, and transfusion requirements. In summary, while preliminary findings are encouraging, rigorous prospective controlled studies are essential before vitamin‐based interventions can be recommended for routine dengue care.

## Author Contributions

Conceptualization: Shah Md Muztahid Hasan Chowdhury, Md. Shafiul Hossen, and Md. Abdul Barek. Data curation: Shah Md Muztahid Hasan Chowdhury, Mahammed Shafin Ul Islam, and Mohammad Safiqul Islam. Formal analysis: Mohammad Safiqul Islam. Funding acquisition: none. Investigation: Mohammad A. Rashid and Mohammad Safiqul Islam. Methodology: Shah Md Muztahid Hasan Chowdhury, Md. Shafiul Hossen, Mohammad Safiqul Islam, and Md. Abdul Barek. Project administration: Shah Md Muztahid Hasan Chowdhury, Arafat Miah, and Mohammad Safiqul Islam. Resources: Mohammad Safiqul Islam and Md. Abdul Barek. Software: Mohammad A. Rashid and Mohammad Safiqul Islam. Supervision: Md. Abdul Barek. Validation: Shah Md Muztahid Hasan Chowdhury, Md. Shafiul Hossen, and Md. Abdul Barek. Visualization: Mohammad A. Rashid, Arafat Miah, and Md. Abdul Barek. Writing – original draft: Shah Md Muztahid Hasan Chowdhury, Md. Shafiul Hossen, Arafat Miah, and Mahammed Shafin Ul Islam. Writing – review and editing: Mohammad A. Rashid, Mohammad Safiqul Islam, and Md. Abdul Barek.

## Funding

The authors received no specific funding for this work.

## Disclosure

The lead author Md. Abdul Barek affirms that this manuscript is an honest, accurate, and transparent account of the study being reported; that no important aspects of the study have been omitted; and that any discrepancies from the study as planned (and, if relevant, registered) have been explained.

## Conflicts of Interest

Mohammed Safiqul Islam is an Editorial Board member of *Health Science Reports* and a co‐author of this article. To minimize bias, they were excluded from all editorial decision‐making related to the acceptance of this article for publication.

## Supporting information


**Supporting Figure 1:** Flow chart of the literature selection process. **Supporting Table 1:** Quality assessment of each included study.

## Data Availability

All data are derived from previously published studies; detailed search strategies and extraction templates will be made available as Supporting Information.

## References

[1] R. Chen and N. Vasilakis , “Dengue—quo tu et quo vadis?,” Viruses 3, no. 9 (2011): 1562–1608, 10.3390/v3091562. 21994796 PMC3187692

[2] O. J. Brady , P. W. Gething , S. Bhatt , et al., “Refining the Global Spatial Limits of Dengue Virus Transmission by Evidence‐Based Consensus,” PLoS Neglected Tropical Diseases 6, no. 8 (2012): e1760, 10.1371/journal.pntd.0001760. 22880140 PMC3413714

[3] S. Bhatt , P. W. Gething , O. J. Brady , et al., “The Global Distribution and Burden of Dengue,” Nature 496, no. 7446 (2013): 504–507, 10.1038/nature12060. 23563266 PMC3651993

[4] B. R. Murphy and S. S. Whitehead , “Immune Response to Dengue Virus and Prospects for a Vaccine,” Annual Review of Immunology 29, no. 1 (2011): 587–619, 10.1146/annurev-immunol-031210-101315. 21219187

[5] J. Jaratsittisin , B. Xu , W. Sornjai , et al., “Activity of Vitamin D Receptor Agonists Against Dengue Virus,” Scientific Reports 10, no. 1 (2020): 10835, 10.1038/s41598-020-67783-z. 32616772 PMC7331731

[6] S. B. Halstead , “Dengvaxia Sensitizes Seronegatives to Vaccine Enhanced Disease Regardless of Age,” Vaccine 35, no. 47 (2017): 6355–6358, 10.1016/j.vaccine.2017.09.089. 29029938

[7] J. Chang , T. M. Block , and J. T. Guo , “Antiviral Therapies Targeting Host ER Alpha‐Glucosidases: Current Status and Future Directions,” Antiviral Research 99, no. 3 (2013): 251–260, 10.1016/j.antiviral.2013.06.011. 23816430 PMC7114303

[8] M. P. Courageot , M. P. Frenkiel , C. Duarte Dos Santos , V. Deubel , and P. Desprès , “α‐Glucosidase Inhibitors Reduce Dengue Virus Production by Affecting the Initial Steps of Virion Morphogenesis in the Endoplasmic Reticulum,” Journal of Virology 74, no. 1 (2000): 564–572, 10.1128/jvi.74.1.564-572.2000. 10590151 PMC111573

[9] A. Khanam , H. Gutiérrez‐Barbosa , K. E. Lyke , and J. V. Chua , “Immune‐Mediated Pathogenesis in Dengue Virus Infection,” Viruses 14, no. 11 (2022): 2575, 10.3390/v14112575. 36423184 PMC9699586

[10] B. E. E. Martina , P. Koraka , and A. D. M. E. Osterhaus , “Dengue Virus Pathogenesis: An Integrated View,” Clinical Microbiology Reviews 22, no. 4 (2009): 564–581, 10.1128/CMR.00035-09. 19822889 PMC2772360

[11] R. Soundravally , P. Sankar , Z. Bobby , and S. L. Hoti , “Oxidative Stress in Severe Dengue Viral Infection: Association of Thrombocytopenia With Lipid Peroxidation,” Platelets 19, no. 6 (2008): 447–454, 10.1080/09537100802155284. 18925513

[12] S. Maggini , A. Pierre , and P. C. Calder , “Immune Function and Micronutrient Requirements Change Over the Life Course,” Nutrients 10, no. 10 (2018): 1531, 10.3390/nu10101531. 30336639 PMC6212925

[13] T. J. Schaefer , P. K. Panda , and R. W. Wolford , “Dengue Fever,” in *StatPearls* (StatPearls Publishing, 2022). 28613483

[14] S. Mukhopadhyay , R. J. Kuhn , and M. G. Rossmann , “A Structural Perspective of the Flavivirus Life Cycle,” Nature Reviews Microbiology 3, no. 1 (2005): 13–22, 10.1038/nrmicro1067. 15608696

[15] A. Bhalla , H. Singh , V. Suri , et al., “ISCCM Position Statement: Management of Severe Dengue in Intensive Care Unit,” Indian Journal of Critical Care Medicine 28, no. Suppl 2 (2024): 42, 10.5005/jp-journals-10071-24748. PMC11369922 39234231

[16] T. Srichaikul , S. Nimmanitaya , N. Artchararit , T. Siriasawakul , and P. Sungpeuk , “Fibrinogen Metabolism and Disseminated Intravascular Coagulation in Dengue Hemorrhagic Fever,” American Journal of Tropical Medicine and Hygiene 26, no. 3 (1977): 525–532, 10.4269/ajtmh.1977.26.525. 869104

[17] R. Kothai and B. Arul , “Dengue Fever: An Overview,” in *Dengue Fever in a One Health Perspective* (IntechOpen, 2020): 28, 10.5772/intechopen.92315.

[18] L. M. Meuren , E. B. Prestes , M. P. Papa , et al., “Infection of Endothelial Cells by Dengue Virus Induces ROS Production by Different Sources Affecting Virus Replication, Cellular Activation, Death and Vascular Permeability,” Frontiers in Immunology 13 (2022): 810376, 10.3389/fimmu.2022.810376. 35185902 PMC8847576

[19] U. C. Chaturvedi , R. Agarwal , E. A. Elbishbishi , and A. S. Mustafa , “Cytokine Cascade in Dengue Hemorrhagic Fever: Implications for Pathogenesis,” FEMS Immunology and Medical Microbiology 28, no. 3 (2000): 183–188, 10.1111/j.1574-695x.2000.tb01474.x. 10865168

[20] M. K. Dash , S. Samal , S. Rout , C. K. Behera , M. C. Sahu , and B. Das , “Immunomodulation in Dengue: Towards Deciphering Dengue Severity Markers,” Cell Communication and Signaling 22, no. 1 (2024): 451, 10.1186/s12964-024-01779-4. 39327552 PMC11425918

[21] F. E. Pehlivan and C. Vitamin , “An Antioxidant Agent,” Vitamin C 2 (2017): 23–35, 10.5772/intechopen.69660.

[22] D. V. Karthik , D. K. Kumar , D. S. Kumar , D. S. Mohammed , D. V. Bharathy , and D. V. Reddy , “Effect of Vitamin E & Vitamin C Supplementation on Thrombocytopenia in Dengue Fever–A Randomized Controlled Trial in Children Aged 2–12 Years,” TPM–Testing, Psychometrics, Methodology in Applied Psychology 32, no. S4 (2025): 1338–1347.

[23] S. D. Langerman and M. Ververs , “Micronutrient Supplementation and Clinical Outcomes in Patients With Dengue Fever,” American Journal of Tropical Medicine and Hygiene 104, no. 1 (2021): 45–51, 10.4269/ajtmh.20-0731. 33258437 PMC7790074

[24] V. Srigade , “Dengue Hemorrhagic Fever: Clinical Efficacy of Vitamin K,” Indian Journal of Child Health 4, no. 1 (2017): 53–56, 10.32677/ijch.2017.v04.i01.014.

[25] D. M. Giraldo , A. Cardona , and S. Urcuqui‐Inchima , “High‐Dose of Vitamin D Supplement Is Associated With Reduced Susceptibility of Monocyte‐Derived Macrophages to Dengue Virus Infection and Pro‐Inflammatory Cytokine Production: An Exploratory Study,” Clinica Chimica Acta 478 (2018): 140–151, 10.1016/j.cca.2017.12.044. 29289621

[26] S. Iqtadar , A. Khan , S. U. Mumtaz , et al., “Vitamin D Deficiency (VDD) and Susceptibility Towards Severe Dengue Fever—A Prospective Cross‐Sectional Study of Hospitalized Dengue Fever Patients From Lahore, Pakistan,” Tropical Medicine and Infectious Disease 8, no. 1 (2023): 43, 10.3390/tropicalmed8010043. 36668950 PMC9866117

[27] T. Bharara , A. Chakravarti , and N. Kapoor , “Correlation of 25‐Hydroxy Vitamin D3 Levels With Dengue Disease Severity–Can Vitamin D Levels Predict Dengue Prognosis?,” International Journal of Infectious Diseases 101 (2020): 260, 10.1016/j.ijid.2020.11.116.

[28] M. G. Traber and J. F. Stevens , “Vitamins C and E: Beneficial Effects From a Mechanistic Perspective,” Free Radical Biology and Medicine 51, no. 5 (2011): 1000–1013, 10.1016/j.freeradbiomed.2011.05.017. 21664268 PMC3156342

[29] E. Agwu , C. Ezihe , and G. Kaigama , “Antioxidant Roles/Functions of Ascorbic Acid (Vitamin C),” in *Ascorbic Acid‐Biochemistry and Functions* (2023), 10.5772/intechopen.110589.

[30] Y. J. Jeong , S. W. Hong , J. H. Kim , et al., “Vitamin C‐Treated Murine Bone Marrow‐Derived Dendritic Cells Preferentially Drive Naïve T Cells Into Th1 Cells by Increased IL‐12 Secretions,” Cellular Immunology 266, no. 2 (2011): 192–199, 10.1016/j.cellimm.2010.10.005. 21074755

[31] S. Mousavi , S. Bereswill , and M. M. Heimesaat , “Immunomodulatory and Antimicrobial Effects of Vitamin C,” European Journal of Microbiology and Immunology 9, no. 3 (2019): 73–79, 10.1556/1886.2019.00016. 31662885 PMC6798581

[32] A. Gęgotek and E. Skrzydlewska , “Antioxidative and Anti‐Inflammatory Activity of Ascorbic Acid,” Antioxidants 11, no. 10 (2022): 1993, 10.3390/antiox11101993. 36290716 PMC9598715

[33] M. S. Ellulu , A. Rahmat , I. Patimah , H. Khaza'ai , and Y. Abed , “Effect of Vitamin C on Inflammation and Metabolic Markers in Hypertensive and/or Diabetic Obese Adults: A Randomized Controlled Trial,” Drug Design, Development and Therapy (2015): 3405–3412, 10.2147/dddt.s83144. 26170625 PMC4492638

[34] E. Candelario‐Jalil , R. S. Akundi , H. S. Bhatia , et al., “Ascorbic Acid Enhances the Inhibitory Effect of Aspirin on Neuronal Cyclooxygenase‐2‐Mediated Prostaglandin E2 Production,” Journal of Neuroimmunology 174, no. 1–2 (2006): 39–51, 10.1016/j.jneuroim.2006.01.003. 16529823

[35] S. R. Pinnell , “Regulation of Collagen Biosynthesis by Ascorbic Acid: A Review,” Yale Journal of Biology and Medicine 58, no. 6 (1985): 553–559. 3008449 PMC2589959

[36] J. M. May and F. E. Harrison , “Role of Vitamin C in the Function of the Vascular Endothelium,” Antioxidants & Redox Signaling 19, no. 17 (2013): 2068–2083, 10.1089/ars.2013.5205. 23581713 PMC3869438

[37] M. S. Uddin , M. S. Millat , P. K. Baral , et al., “The Protective Role of Vitamin C in the Management of COVID‐19: A Review,” Journal of the Egyptian Public Health Association 96, no. 1 (2021): 33, 10.1186/s42506-021-00095-w. 34894332 PMC8665316

[38] R. M. L. Colunga Biancatelli , M. Berrill , J. D. Catravas , and P. E. Marik , “Quercetin and Vitamin C: An Experimental, Synergistic Therapy for the Prevention and Treatment of SARS‐CoV‐2 Related Disease (COVID‐19),” Frontiers in Immunology 11 (2020): 550247, 10.3389/fimmu.2020.01451. PMC7318306 32636851

[39] N. Valero , J. Mosquera , S. Alcocer , E. Bonilla , J. Salazar , and M. Álvarez‐Mon , “Melatonin, Minocycline and Ascorbic Acid Reduce Oxidative Stress and Viral Titers and Increase Survival Rate in Experimental Venezuelan Equine Encephalitis,” Brain Research 1622 (2015): 368–376, 10.1016/j.brainres.2015.06.034. 26168898

[40] W. Li , N. Maeda , and M. A. Beck , “Vitamin C Deficiency Increases the Lung Pathology of Influenza Virus–Infected Gulo^−/−^ Mice,” Journal of Nutrition 136, no. 10 (2006): 2611–2616, 10.1093/jn/136.10.2611. 16988135

[41] Y. Cai , Y. F. Li , L. P. Tang , et al., “A New Mechanism of Vitamin C Effects on A/FM/1/47 (H1N1) Virus‐Induced Pneumonia in Restraint‐Stressed Mice,” BioMed Research International 2015, no. 675149 (2015): 675149, 10.1155/2015/675149. 25710018 PMC4331320

[42] A. Carr and S. Maggini , “Vitamin C and Immune Function,” Nutrients 9, no. 11 (2017): 1211, 10.3390/nu9111211. 29099763 PMC5707683

[43] K. Ramalingam , C. S. Varghese , C. Elias , G. M. Mathew , and A. Balasubramanian , “A Retrospective Study on the Effect of Vitamin C in the Management of Dengue Fever in Three Different States of India,” International Journal of Research in Pharmaceutical Sciences 10, no. 4 (2019): 2670–2673, 10.26452/ijrps.v10i4.1525.

[44] S. Ahmed , J. L. Finkelstein , A. M. Stewart , et al., “Micronutrients and Dengue,” American Society of Tropical Medicine and Hygiene 91, no. 5 (2014): 1049–1056, 10.4269/ajtmh.14-0142. PMC4228873 25200269

[45] Country Office for India, World Health Organization , National Guidelines for Clinical Management of Dengue Fever (WHO Country Office for India, 2015), https://iris.who.int/handle/10665/208893.

[46] S. Kalayanarooj , “Clinical Manifestations and Management of Dengue/DHF/DSS,” Tropical Medicine and Health 39, no. 4SUPPL (2011): S83–S87, 10.2149/tmh.2011-s10. PMC3317599 22500140

[47] M. J. Shearer and P. Newman , “Recent Trends in the Metabolism and Cell Biology of Vitamin K With Special Reference to Vitamin K Cycling and MK‐4 Biosynthesis,” Journal of Lipid Research 55, no. 3 (2014): 345–362, 10.1194/jlr.r045559. 24489112 PMC3934721

[48] World Health Organization , National Guideline for Clinical Management of Dengue 2022 (Democratic Republic of Timor‐Leste, 2022).

[49] Ministry of Health, Nutrition, and Indigenous Medicine, Sri Lanka. *National Guidelines: Management of Dengue Fever and Dengue Haemorrhagic Fever in Children* (Epidemiology Unit, 2012), https://medicine.kln.ac.lk/depts/publichealth/Fixed_Learning/dengue%20guidelines/children.pdf.

[50] K. Alagarasu , “Immunomodulatory Effect of Vitamin D on Immune Response to Dengue Virus Infection,” Vitamins and Hormones 117 (2021): 239–252, 10.1016/bs.vh.2021.06.001. 34420583

[51] K. Alagarasu , R. V. Bachal , A. B. Bhagat , P. S. Shah , and C. Dayaraj , “Elevated Levels of Vitamin D and Deficiency of Mannose Binding Lectin in Dengue Hemorrhagic Fever,” Virology Journal 9, no. 1 (2012): 86, 10.1186/1743-422x-9-86. 22559908 PMC3413536

[52] J. F. Arboleda Alzate , I. A. Rodenhuis‐Zybert , J. C. Hernández , J. M. Smit , and S. Urcuqui‐Inchima , “Human Macrophages Differentiated in the Presence of Vitamin D3 Restrict Dengue Virus Infection and Innate Responses by Downregulating Mannose Receptor Expression,” PLoS Neglected Tropical Diseases 11, no. 10 (2017): e0005904, 10.1371/journal.pntd.0005904. 29020083 PMC5653353

[53] H. Loke , D. Bethell , C. X. T. Phuong , et al., “Susceptibility to Dengue Hemorrhagic Fever in Vietnam: Evidence of an Association With Variation in the Vitamin D Receptor and Fc Gamma Receptor IIa Genes,” American Journal of Tropical Medicine and Hygiene 67, no. 1 (2002): 102–106, 10.4269/ajtmh.2002.67.102. 12363051

[54] E. Villamor , L. A. Villar , A. Lozano , V. M. Herrera , and O. F. Herrán , “Vitamin D Serostatus and Dengue Fever Progression to Dengue Hemorrhagic Fever/Dengue Shock Syndrome,” Epidemiology and Infection 145, no. 14 (2017): 2961–2970, 10.1017/s0950268817002059. 28903788 PMC9152741

[55] D. Saha , S. Mandal , S. Roy , et al., “Micronutrients and Dengue Fever: Exploring Their Role in Effective Management Strategies,” Current Nutrition & Food Science 21, no. 6 (2025): 702–712, 10.2174/0115734013340202241108062720.

