# Supplementary material for: Protective Role of Vitamins C, D, and K in the Management of Dengue: A Narrative Review to Combat the Dengue Outbreak
Source: Health Sci Rep. 2026 Feb 17;9(2):e71823. doi: 10.1002/hsr2.71823 (PMC12914079; doi:10.1002/hsr2.71823)
Supplement: Supplementary file 1 — Supporting Figure 1: Flow chart of the literature selection process. Supporting Table 1: Quality assessment of each included study. [file HSR2-9-e71823-s001.docx]

Shortlisted after the elimination of duplicate study (n = 158)

Articles published before March 2025 were identified using the terms dengue, vitamin C, vitamin D, vitamin K, dengue management and treatment etc. from Google Scholar, PubMed, EMBASE, Cochrane Library, and Web of Science (n = 232)

Screening of title and abstract (n = 110)

Full-text review (n = 73)

Studies included

(n = 55)

Duplicates eliminated

(n = 74)

Not related to the topic (n = 48)

Studies excluded (n = 37)

- Irrelevant to the study
- Lack of full-text access

Excluded after full text review (n = 18)

- Reviews, commentaries, letter to editors

Identification

Screening

Eligibility

Included

**Supplementary Figure 1:** Flow chart of the literature selection process

| **Supplementary Table 1:** Quality assessment of each included study | | | |
| --- | --- | --- | --- |
| **Questions/ Checklists** | **Total included studies (N = 55)** | | |
|  | **Yes** | **No** | **Unclear** |
| Was there a clear statement of the aims of the research? | 55 |  |  |
| Were the inclusion criteria appropriate for the review question/ statement/ aim? | 55 |  |  |
| Is a qualitative methodology appropriate? | 55 |  |  |
| Was the research design appropriate to address the aims of the research? | 54 |  | 1 |
| Were the sources and resources used to search for studies adequate? | 53 |  | 2 |
| Were there methods to minimize errors in data extraction? | 50 |  | 5 |
| Was the likelihood of publication bias assessed? | 49 |  | 6 |
| Have ethical issues been taken into consideration? | 53 | 1 | 1 |
| Was the data analysis sufficiently rigorous? | 55 |  |  |
| Is there a clear statement of findings? | 55 |  |  |
